# Circular Letter to the Profession of Texas—by Committee on Legislation

**Published:** 1890-10

**Authors:** 


### Society Notes.

### CIRCULAR LETTER TO THE MEDICAL PROFESSION OF THE STATE OF TEXAS.

DEAR DOCTOR:—The total neglect of Preventive Medicine in our State, through the failure of previous Legislatures to frame enactments on Sanitation, notwithstanding the earnest and repeated prayers of the profession, and the wide-felt necessity for such legislation, has placed our great and wealthy State in a contrast, anything but honorable, with the position occupied in this matter, by no less than thirty-five States which have created Boards of Health, charged with the care of the public health, and with the care of admitting none but competent physicians to practice medicine and surgery within their limits. Nay more, every death from preventable disease now taking place in Texas, is another proof of culpable neglect on the part of past Legislatures.

Believing the Legislature shortly to be elected, will be more accessible to appeals to their reason, and to their sense of duty, as also to the growing interest taken by the people at large in sanitary measures, the Committee on Legislation of the Texas State Medical Association, earnestly and most emphatically solicit your active co-operation in formulating the wishes of the people, and of the medical profession in a petition to the two Houses, copies of which will be furnished you, that the action taken may be uniform and systematic.

To this petition it would be well to obtain the signatures of the candidates for leglslative honors in your county and district, and in addition, as many signatures as you can obtain in your neighborhood. Doctor, let each physician be earnest in this matter, in season and out of season; let each one be an apostle of humanity and of progress, and the reproach will pass away from us, that while State provision is promised for the health of the cattle of our State, human life continues to be the cheapest commodity in Texas, and she still continues to furnish a sure asylum for all quacks and charlatans, driven from more enlightened States by their laws regulating the practice of medicine, excluding the incompetent.

Preventive or State Medicine and Sanitation, an important branch of it, are not, as is generally supposed, of modern origin, although practically it had become obsolete in modern times, until early in this century, it became a living question, principally through the advocacy of Chadwick, Simon and others in Great Britain; and later in the United States, thanks to the labors of Bowditch, of Boston, and N. S. Davis, of Chicago, supplemented by a host of others, until it has become the most important question of the day, as affecting the public health and the conservation of life.

The fact that thirty-five States of the Union, have created boards of health, one of them, Florida, having called a special session of its Legislature for that purpose, suffices to show the paramount importance of sanitary supervision, in the estimation of statesmen.

What has been done in the great, the wealthy, the Empire State of Texas? Virtually nothing; save for a costly quarantine establishment and a law, which remains a dead letter, the public health has been left to take care of itself, the unsystematized efforts of individual physicians have remained nugatory for want of concert of action and of organization.

The Mosaic Law consisted in great part of regulations of personal and public hygiene.

The Mahometan Code contains one feature worthy of imitation in the daily ablutions prescribed as a part of their religious ritual. The empress city of Rome, seated on her seven hills, could point to no grander features in her domestic polity, than the aquaducts and baths which ministered to the health and comfort of her poorest citizens, and wherever her legions bore their conquering eagles, like structures arose, and remain to this day, enduring monuments of the enlightened civilization of the mistress of the world.

It is a crying shame that outside of a few cities, a human being is consigned to the tomb, with no more recognition by law than one of the lower animals, and with no more authentic record of his existence than if he had never been.

The facilities afforded to secret crime by this want of legal record, are too obvious to require discussion. The same arguments apply to the registration of births as of deaths. The very existence of a human being, involving it may be, vast interests, cannot be proven in Texas by law by any but oral testimony, which, in the lapse of a generation or two, vanishes forever! Common sense, as well as judicial reason, dictates that of every birth and of every death occurring in the vast territory of our State, there should remain an authentic and lasting record! The record of marriages is already provided for by the law, and with a record of the births and deaths would constitute a complete civil status of every one born and dying in the State.

The spread of Asiatic cholera in Europe, Asia and Africa, admonishes us that we should be up and doing; that the pestilence which walketh in darkness, and cometh like a thief in the night, may ere long be at our gates, and find us unprepared. Yet we earnestly wish to impress on the people, that the deaths from preventable disease, far outnumber those from epidemics, and the continual daily dropping of lives, which might have been saved by sanitary precaution, swell the mortuary records and increase the death rate more, far more, than the direst visitations of cholera, of yellow fever, or of the much dreaded small-pox, which latter, a law enforcing vaccination would soon eradicate from our land, as has been done in other countries, notably in Denmark.

It has never been fully comprehended by the Committee, why the protection to life and health afforded by local boards of health should be restricted to the residents in cities, to the exclusion of the people inhabiting the rural districts. The children of the farmer are as dear to him, as those of the denizens of cities can be to them, and are entitled to the same protection, neither more nor less, and in their name and in that of equal justice, we demand it at the hands of the legislative branch of our State government.

It has been demonstrated by the Michigan Board of Health that the services rendered by rural boards of health, have been even more marked than in cities. It has been computed by Sir T. Spencer Wells, that the pecuniary loss to Great Britain from sickness, amounts per annum in the aggregate to £20,000,000 or $100,000,000.

In that country, thanks to the admirable health system, dating from 1838, forming an integral branch of the government, as it should be here, the death rate has diminished four per thousand per annum, a saving of 12,000 lives a year. Taking this basis of calculation in Texas, with a population of 3,000,000, the pecuniary loss per annum from preventable sickness, would amount to one-tenth that of Great Britain, or $10,000,000, and what figures shall represent the care, the anxiety the mortal agonies of watchers by the couch of the victims of preventable disease, all of which, we repeat, are evidences of the most culpable neglect on the part of the legislators, who failed to perform their most important, their most urgent duty, in the protection of the people committed to their charge, in their lives and health. How pitiful does the economical, nay, the penurious considerations of the legislative body appear in the light of the above facts. Out on such economy which sacrifices human life to political calculations! The statesman who shall rise above them, will live in the memory of a grateful people, and be crowned with a wreath such as conqueror never wore.

In the palmy days of the Roman Republic, the civic crown of Oak Leaves which adorned the man who saved the life of a Roman citizen, ranked above the laurel wreath of her Cæsars, her Pompeys, her Antonys. Might it not be well to revive some of the practices as well as the theories of the great Republic of old.

We have in the foregoing pages, touched on but a few of the crying evils due to the question of the public health having been totally ignored by the law-makers of Texas. We believe that a new era is at hand, that public opinion has been educated to that point, that it will demand appropriate legislation; we believe that the members of the new legislature are now alive to the gravity of that question, and that an appeal to them by the great mass of the people will reach willing ears. Hence the movement which the Committee on Legislation seeks to inaugurate. The remedy for the evils enumerated above, and for many others, is to be found in a State Board of Health appointed under the provisions of the Constitution, having cognizance of all matters affecting the health of the people, and constituting a branch of the State government as it does in many other countries, with longer experience than ours.

The appeals on behalf of the people of our State by the medical profession, have heretofore been met by arguments of economy, urging the cost to the State. The political economy which ignores the safety of the people, to save them a few thousand dollars per annum, is not of the highest order, and elsewhere would hardly be deemed respectable.

The central board of health should have under its supervision and direction, a board in each county, of say three members, one of whom should be the health officer, and be charged with the duty of receiving registration of all births in the county, as also of all deaths, interment without a permit from the health officer being declared an offense punishable by fine. In extensive counties the members of the county board could act as deputy health officers to save inconvenience to those having to make declarations of births or deaths in their families.

In this manner an absolutely correct statistical statement could be prepared of the births, marriages (the latter obtained from the county clerk under the present law), and deaths, with the ratio of increase by births, and the ratio of mortality to the population. This would be, to use a commercial illustration, taking stock of our people, and a correct and invaluable guide for sanitary measures, as showing the prevailing and more fatal diseases throughout the State. Furthermore, the supervision of local health reports by a central board, would compel correctness in their details and their results, aud would prevent the egregious lying of figures, notwithstanding the proverb; and in nothing is this more conspicuous than in sanitary reports, based on incorrect premises, to the advantage of certain localities. In the event of an epidemic of contagious or infectious disease breaking out, these local boards would constitute the machinery necessary to isolate, and if possible to limit the ravages of disease; at present nothing of this exists, and if Asiatic cholera should reach our shores, a perfectly possible contingency, all would be hurry, alarm, confusion and inefficiency. A single case of small-pox may inaugurate a reign of terror to our families, dislocating the relations of commerce and of business of every description, and who shall stay it? The increased rapidity of travel and its vast extension may lead to an invasion of the interior by yellow fever, as happened in 1867, and who shall cope with it?

The creation of a Central and Local Board of Health would add a mere fraction of one cent on the hundred dollars of the assessed value of property in the State, and would be the most useful expenditure ever authorized. A very small supplementary tax levied by each county in the ratio of its population would suffice, and would save many lives.

Ere another Legislature can assemble two years must elapse. Now is the accepted time. Ere the expiration of two years more the pestilence may have swept over our land, which God forbid, leaving thousands of desolate homes and bereaved families to mourn the blind infatuation which left all to the care of Providence.

Providence endowed man with the intelligence which is his attribute, that he might help himself, and then Providence will smile on his strenuous efforts.

We implore the people to lay this matter to heart; it interests every human being in the State, not only in his own personality, but as affecting those dearer than self. It is a duty incumbent on every individual to unite in demanding of our Legislature prompt and efficient action.

In advocating the creation of a State Board of Health, a most important and much needed object contemplated by the Committee on Legislation is an efficient law regulating the practice of medicine in all its branches, which will put to flight the hordes of impostors now preying on the ignorance of our people, who have no means of discriminating between the legally qualified and competent physician and the impudent quack. The law desired by the Committee would embody the following as its principal features:

1. The State Board of Health, composed of physicians nominated by the State Association and appointed by the Governor, shall constitute a Board of Examiners, before whom all persons intending to practice medicine or any of its branches, except dentistry, in the State, shall present themselves for examination.

2. A general register of the legally qualified practitioners of medicine and surgery of the State of Texas, of which the State Board of Health shall be the custodian. This register shall be open to all who at the date of the passage of the law shall have qualified under the laws now or heretofore in force, and to those qualifying by examination under the new law. The value of this register as a means of guiding the people in their choice of physicians, is self-evident.

3. Anyone claiming to practice medicine, whose name is not borne on the register, shall not be competent to recover at law payment for medical services; he shall not be exempt from militia and jury duty; he shall not be admitted to give expert testimony in any court of law, or to give a medical certificate to be used in litigation; he shall not be eligible to any medical appointment, State, county or municipal, or as a medical examiner for any life insurance or benefit society doing business in the State; and he shall be subject to punishment by fine or imprisonment, or both, for illegal practice of medicine, on conviction thereof in a court of competent jurisdiction. The necessity for amending the present apology for a law regulating the practice of medicine, is pressing to every one who has given the subject a thought. The remedy is in the hands of the people; let them make known to the Legislature their will in the matter in language not to be misunderstood, and the desired reform will be made, and the stigma will no longer attach to our State that while the practice of dentistry and pharmacy have been regulated by law, and the health of our cattle is promised protection, yet the health of her sons and daughters, the true wealth of a commonwealth, has been totally neglected and no adequate means provided for its conservation.

Geo. Cupples, M. D.,
Thos. D. Wooten, M. D.,
J. W. McLaughlin, M. D.,
T. J. Tyner, M. D.,
—————, M. D.,
—————, M. D.,
Committee on Medical Legislation, T. S. M. A.

---

The above address from the Committee on Medical Legislation, to the number of 6000, has been struck off in pamphlet form and a copy will be sent to every physician in Texas, as well as to a large number of prominent laymen. Each letter was accompanied by the following note; and a petition, of which the following is a copy, was enclosed in each:

San Antonio, October 16, 1890.

Doctor:—Counting on your hearty approval of the objects of the present move for Medical Reform, and your co-operation in securing it, we venture to suggest that you interest as many influential members as possible of your community in obtaining signatures to the petition to the Legislature, as also the local press, and that you return the petition by the 31st of December, with as many signatures as can be procured.

Address the petition to me at San Antonio. Fraternally yours,

GEO. CUPPLES,
Chairman Com. on Medical Legislation.

